# DJ-1 alleviates high glucose-induced podocyte injury via activating ERK1/2 signaling

**DOI:** 10.1371/journal.pone.0346714

**Published:** 2026-04-17

**Authors:** Xiaochen Tian, Jing Li, Tao Han, Leisheng Zhang, Ping Lu, Yusheng Liu

**Affiliations:** 1 Shandong University, Jinan, China; 2 The Fourth People’s Hospital of Jinan, Shandong Second Medical University, Jinan, China; 3 Internal Medicine of Jinan Municipal Government Hospital, Jinan, China; 4 Community Management Department of The Fourth People’s Hospital of Jinan, Jinan, China; 5 Science and Technology Innovation Center, Shandong Provincial Key Medical and Health Laboratory of Blood Ecology and Biointelligence, Jinan Key Laboratory of Medical Cell Bioengineering, Cardio-cerebrovascular Disease Hospital of Jinan, China; 6 The Second Hospital of Shandong University, Jinan, China; Universidade de Sao Paulo, BRAZIL

## Abstract

Diabetic nephropathy (DN) is a major diabetic complication, and while DJ-1 has been shown to mitigate renal ischemia/reperfusion injury, its role in high glucose-induced podocyte damage remains unclear. This study aimed to investigate the function and mechanism of DJ-1 in high glucose-induced injury of human podocyte cells (HPCs). Using RNA-seq analysis, we identified that high glucose broadly affected multiple signaling pathways related to cell growth, death, and signal transduction. Notably, DJ-1 expression was downregulated under high glucose conditions. Overexpression of DJ-1 significantly attenuated high glucose-induced HPC apoptosis. Mechanistically, DJ-1 promoted the phosphorylation of ERK1/2 and facilitated the nuclear translocation of p-ERK1/2. This activated the ERK1/2 pathway and upregulated the expression of NF-κB p65 and AP-1, thereby suppressing HPC apoptosis under high glucose conditions. In summary, our findings reveal that DJ-1 protects against high glucose-induced HPC injury by activating the ERK1/2 pathway and enhancing NF-κB p65 and AP-1 expression, providing new insights into the molecular mechanisms of DJ-1 in diabetic nephropathy.

## Introduction

Diabetic nephropathy (DN) is a major microvascular complication of diabetes, and podocyte apoptosis is a critical event in its pathogenesis [1,2]. A hyperglycemic milieu induces oxidative stress and inflammatory responses, leading to renal cell damage [3–5]. Patients with DN often have hypertension and renal vasoconstriction. Long-term exposure to high glucose levels results in the formation of advanced glycation end products (AGEs), which accumulate in the kidneys and cause structural and functional damage [6]. Furthermore, patients with DN often have hypertension and renal vasoconstriction, resulting in renal injury by inducing apoptosis in renal tubular cells [7]. However, the precise molecular mechanisms underlying high glucose-induced podocyte injury remain incompletely understood.

The MAPK signaling pathway, particularly ERK1/2, plays a context-dependent role in cell survival and apoptosis [8]. ERK1/2 exhibits a dual effect on cell apoptosis, promoting it under certain conditions while inhibiting it in others [9,10]. Its effect depends on factors such as cell type, stimulus, and signaling pathways [8,11,12]. ERK1/2 has been found to exert anti-apoptotic effects by phosphorylating anti-apoptotic proteins such as Bcl-2, which inhibits the decrease in mitochondrial membrane potential and reduces the release of cytochrome C [11,13]. In diabetic conditions, ERK1/2 signaling is often dysregulated, but its specific function and regulation in podocytes under high glucose stress is complex and requires further elucidation.

DJ-1 (PARK7) is a multifunctional protein recognized for its protective roles against oxidative stress and in cellular stress responses [14,15]. Through its previously described functions, this protein has also been repeatedly implicated in combating renal ischemia/reperfusion (IR) damage, which is associated with high levels of oxidative stress and underlies several diseases such as stroke, cancer, and diabetes [14–16]. However, its function in diabetic kidney disease, especially concerning podocyte survival, is largely unknown. Given the shared features of oxidative stress in different forms of renal injury, we hypothesized that DJ-1 might confer protection in podocytes against high glucose-induced apoptosis.

In this study, we aimed to investigate the role of DJ-1 in high glucose-stimulated human podocyte cells (HPCs) and to delineate the underlying mechanisms. We found that high glucose significantly downregulated DJ-1 expression. Through gain-of-function studies, we demonstrated that DJ-1 overexpression alleviated high glucose-induced HPC apoptosis. Mechanistically, we discovered that DJ-1 promotes the phosphorylation and nuclear translocation of ERK1/2, leading to the subsequent upregulation of the transcription factors NF-κB p65 and AP-1, which collectively mediate the protective effects. Our findings reveal a novel DJ-1/ERK axis that protects HPCs from high glucose-induced injury, providing new insights into potential therapeutic strategies for DN.

## Materials and methods

### 1. Cell culture and treatments

The conditionally immortalized HPCs used in this study were a temperature-sensitive SV40 T-antigen immortalized cell line (provided by Prof. Fan Yi, Shandong University), and all experiments in this study were performed using fully differentiated HPCs under non-permissive culture conditions. Proliferative (permissive) conditions: HPCs were cultured in RPMI1640 medium containing 10% FBS, 1% penicillin–streptomycin at 33°C (the permissive temperature for SV40 T-antigen activity) in a 5% CO_2_ incubator to maintain logarithmic proliferation. Differentiation (non-permissive) conditions: For experimental use, HPCs were seeded in culture plates at a density of 1 × 10^4^ cells/cm², and the cells were cultured at 37°C (the non-permissive temperature, which inactivates SV40 T-antigen and initiates differentiation) for 10–14 days. The medium was changed every 2 days during differentiation.

For mannitol control, the HPCs were cultured with the medium containing 5.5 mM D‐glucose and 24.5 mM mannitol (MG) for 48 h. For low D‐glucose treatment, the HPCs were cultured with the medium containing 5.5 mM D‐glucose (LG) for 48 h. For high D‐glucose treatment, the HPCs were cultured with the medium containing 30 mM D‐glucose (HG) for 48 h. For all DJ-1 overexpression-related experiments, the 1 μg/mL empty Flag-pcDNA.3.1 vector was transfected unless the transfection of 1 μg/mL DJ-1- Flag-pcDNA.3.1.

### 2. Recombinant plasmid construction, siRNA, and cell transfection

The CDS region of the cDNA of human DJ-1 was amplified by PCR from the cDNA of HPCs using specific primers and inserted into the NheI and BamHI sites in the Flag-pcDNA.3.1 vector (Sangon Biotech, Shanghai, China). The HPCs were plated in 6-well plates, and the transfection was conducted when the cells reached 70% confluence according to the specification (Lipofectamine 3000, L3000001, Invitrogen, USA). After 48 h, the cells were obtained for qRT-PCR and Western blotting. DJ-1 forward primer: GCTAGCGGCTTCCAA AAGAGCTCTGG, reverse primer: GGATCCCTAAGTCTTTAAGAACAAG. All siRNAs were synthesized by Sangon Biotech (Shanghai, China). DJ-1 siRNA#1: 5′-CTCCACTTGTTCTTAAAGA-3′, siRNA#2: 5′-GGTTTTGGAAGTAAAGTTATT-3′, siRNA#3: 5′-AGG CGC GGC TGCAGT CTT TAA-3′.

### 3. Western blotting analysis

RIPA lysis buffer (Solarbio, China) was used to extract cell proteins. SDS–PAGE was performed to separate the proteins, and then transferred to a nitrocellulose membrane. The membrane was blocked with 8% nonfat milk and incubated with the primary antibody overnight at 4 °C, and then incubated with the secondary antibody for 1 h at 37 °C. The membrane was visualized with an ECL Western blotting Detection Kit from GE Healthcare (Waukesha, WI, USA). Antibodies: Anti-DJ-1 (11681–1-AP, Proteintech) and anti-cleaved caspase-3 (25128–1-AP, Proteintech); anti-Bcl-2 (AB1722; Millipore), anti-NF-κB P65 (10745–1-AP, Proteintech), anti-AP-1/c-Fos (66590–1-Ig, Proteintech), anti-ERK1/2 (11257–1-AP, Proteintech), anti-p-ERK1/2 (Thr202/Tyr204) (28733–1-AP, Proteintech), anti-mouse IgG (HRP-linked, SA00001−1, Proteintech), anti-rabbit IgG (HRP-linked, SA00001−2, Proteintech), and anti-β-actin (20536–1-AP, Proteintech).

### 4. Quantitative real-time polymerase chain reaction (qRT-PCR)

Total RNA was isolated from HPCs according to the TRIzol (TRIeasy® LS Total RNA Extraction Reagent, 19201ES60, Yeasen, Shanghai, China) extraction method and reverse transcribed using HiFair III 1st Strand cDNA Synthesis SuperMix from Yeasen (11139ES60, Shanghai, China). Quantitative PCR was performed by using Hieff qPCR SYBR Green Master Mix from Yeasen. The 2^-ΔΔCt^ method was used to analyze the Fold changes in gene expression. GAPDH was used as the internal control for normalization. DJ-1 Forward primer: 5’-CCTGGTGTGGGGCTTGTAA-3’ Reverse primer: 5’-ACCACATCACGGCTACACTG-3’.

### 5. Cell viability measurement

Podocyte viability was measured by using the cell counting kit-8 assay (CCK-8) (C0037, Beyotime, China) and EdU assay (C10310-3, RiboBio, Guangzhou, China). For the CCK-8 assay, HPCs were cultured in 96-well plates (2000 cells/well), and treated with D-glucose, Temuterkib (1μM) (HY-101494, MCE, NJ, USA), or transfected with DJ-1-Flag-pcDNA3.1 (1 μg/mL), CCK-8 assays were used to assess cell viability in 48 h after treatment. In addition, the 5-ethynyl-2’-deoxyuridine incorporation assay was performed by using a Cell-LightTM EdU imaging detection kit according to the manufacturer’s instructions.

### 6. Annexin V-FITC/PI apoptosis assay

Annexin V-fluorescein isothiocyanate/propidium iodide (Annexin V-FITC/PI) apoptosis detection kit (HY-K1073, MCE, NJ, USA) was carried out to measure HPC apoptosis. The procedure was done in accordance with the manufacturer’s instructions. HPCs were resuspended in precooled binding buffer. 10 μL of Annexin V stock solution was added to cells and mixed. The mixture was incubated at 4 °C for 30 min. Subsequently, 10 μL of PI was added and incubated for 8 min. Then, HPCs were measured with a FACS analyzer (BD Biosciences, San Jose, CA, USA).

### 7. RNA-seq

Total RNA was extracted from HPCs treated with 5.5mM (LG), 15mM (MG) and 30 mM (HG) D-glucose for 24 h, and was then subjected to RNA-seq analysis by Shanghai Majorbio Bio-pharm Technology Co. Ltd. The data were analyzed on the free online platform Majorbio Cloud Platform (www.majorbio.com). Genes with an FDR-adjusted *p* value < 0.05 and |log2 fold change| ≥ 1.5 were considered significantly differentially expressed. Heatmaps of the differentially expressed genes (DEGs) were generated using unsupervised hierarchical clustering with the pheatmap package (v1.0.12). GO and KEGG functions with a *P* value (*p* adjust) < 0.05 by Fisher’s exact test were considered significantly enriched.

### 8. Statistical Analyses

All data were expressed as the means ± SD. All experiments were performed at least three times independently. The data were analyzed by using GraphPad Prism 8 (GraphPad Software Inc.) One-way ANOVA or Two-way ANOVA was used for comparisons between groups, as appropriate for the experimental design. Statistical significance was considered when *p* values < 0.05. *p < 0.05; **p < 0.01; ***p < 0.001.

## Results

### RNA-seq analysis reveals that high glucose treatment affects the function of HPCs widely

**Fig 1:**
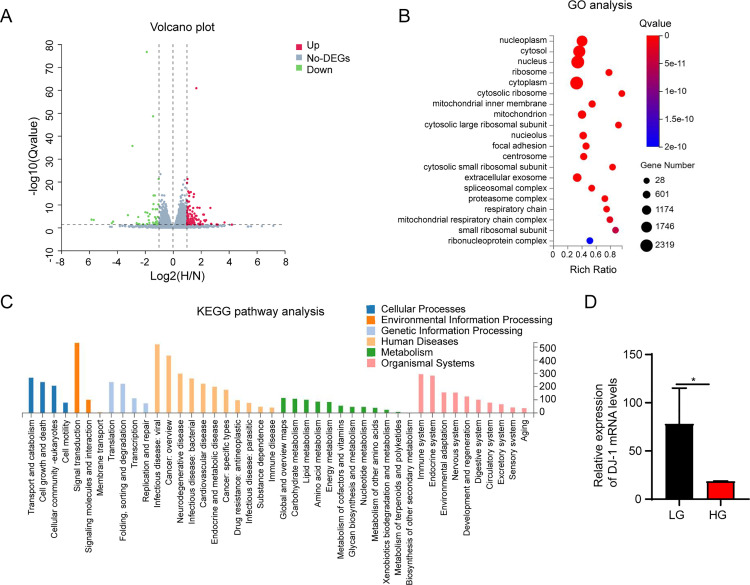
RNA-seq analysis reveals that high glucose treatment affects the function of HPCs widely. **(A)** Volcano plot indicating the differently expressed genes identified by RNA-seq analysis in HPCs treated with High D-glucose (30 mM) and normal D-glucose (5.5 mM). **(B)** GO functional enrichment analysis was performed to analyze the functions of glucose-regulated genes. **(C)** KEGG enrichment analysis was performed to analyze the glucose-regulated pathways. **(D)** The mRNA levels of DJ-1 in were analyzed according to the RNA-seq profile.

Although it has been reported that hyperglycemic environment leads to the damage of kidney cells and further exacerbates kidney injury [6], the mechanisms by which high glucose regulates kidney injury are unclear. We treated the HPCs with 5.5 mM (low dose, LG) and 30 mM (high dose, HG) D-glucose for 48 h, respectively. Then, the treated HPCs were used to perform RNA-seq analysis. RNA-seq analysis revealed that high glucose regulated the expression of 7671 genes, including 3891 up-regulated genes and 3780 down-regulated ones (|log2 fold change| ≥ 1.5, *p* adjust <0.05, Fig 1A). Furthermore, the gene ontology (GO) analysis revealed that high glucose treatment regulated genes related to the nucleoplasm, cytosol, mitochondrion, and other cellular components (Fig 1B). KEGG pathway analysis indicated that high glucose treatment affects several biological aspects including cell growth and death, signal transduction, cell motility, cellular community, signaling molecules and interaction, and cancer-related pathways (Fig 1C), suggesting that high glucose has wide effects on HPCs. Next, we tried to find out the key factor that regulates apoptosis pathway in the high glucose conditions. Importantly, we found that DJ-1, which plays a crucial role in various biological processes including cell protection, was down-regulated in the high glucose-treated HPCs (Fig 1D), suggesting that DJ-1 may be negatively correlated with the high glucose-induced podocyte injury.

### DJ-1 attenuates the high glucose-induced apoptosis of HPCs

**Fig 2:**
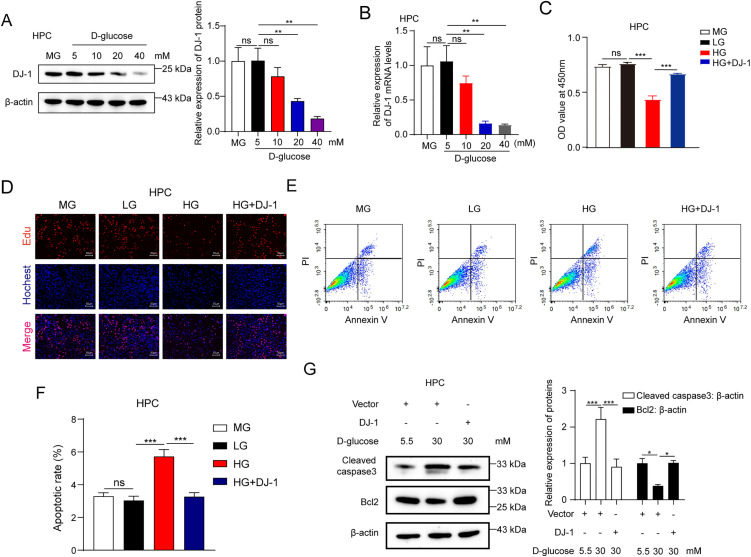
DJ-1 attenuates the high glucose-induced apoptosis of HPCs. **(A)** Representative Western blot images and quantitative statistical bar graphs of protein expression levels of DJ-1 in HPCs treated with mannitol (MG) or D-glucose were analyzed by Western blotting analysis and Image J software, respectively. **(B)** The mRNA levels of DJ-1 in HPCs treated with MG or D-glucose were analyzed by qRT-PCR analysis. **(C)** The cell viability was measured by CCK-8 assays in HPCs treated with MG, LG, HG and HG + DJ-1, respectively. **(D)** The cell viability was analyzed by EdU assays in HPCs treated with MG, LG, HG and HG + DJ-1, respectively. **(E)** The cell apoptosis was analyzed by Annexin V-FITC/PI apoptosis assay in HPCs treated with MG, LG, HG and HG + DJ-1, respectively. **(F)** The apoptotic rate of HPCs treated with MG, LG, HG and HG + DJ-1 was analyzed by GraphPad Prism 8 software. **(G)** Representative Western blot images and quantitative statistical bar graphs of protein expression levels of cleaved caspase 3 and Bcl2 in HPCs treated with LG, HG and HG + DJ-1 were analyzed by Western blotting analysis and Image J software, respectively. MG: mannitol, LG: 5.5mM D-glucose, MG: 15mM D-glucose, HG: 30mM D-glucose, DJ-1: transfected with 1 μg/mL DJ-1-Flag-pcDNA3.1 plasmid. Data are shown as mean ± SD and representative of three independent experiments. *p < 0.05; **p < 0.01; ***p < 0.001.

Next, we explored the function of DJ-1 in HPCs under the condition of high glucose. We first verified the effect of high glucose on the expression of DJ-1. By using Western blotting and qRT-PCR analyses, we found that the expression of DJ-1 was significantly down-regulated as the increase of glucose dose in HPCs in both mRNA and protein levels (Fig 2A and B). Furthermore, we tried to explore the function of DJ-1 on HPCs in the condition of high glucose. We performed a CCK-8 assay to analyze the effect of DJ-1 on cell viability. As shown in Fig 2C, OD450 of high glucose treatment fell by ≈ 40% at 48 h (LG 0.76 ± 0.02 vs HG 0.44 ± 0.04, P < 0.0001), 95% CI = [0.2176, 0.4297]. DJ-1 over-expression rescued ≈ 70% of this loss (HG + DJ-1 0.67 ± 0.01), 95% CI for the HG + DJ-1 vs HG difference = [0.1246, 0.3367]. Consistently, EdU assay further demonstrated that overexpression of DJ-1 could increase the survival capacity of HPCs under high glucose conditions (Fig 2D), suggesting that DJ-1 could significantly suppress the high glucose-induced viability inhibition of HPCs. Next, we analyzed the function of DJ-1 on HPC apoptosis under high glucose conditions. We found that the Annexin-V/PI-positive fraction declined from 5.7% ± 0.5% (HG) to 3.3% ± 0.3% with DJ-1 OE, a 42% relative reduction (P = 0.001), 95% CI = [−3.237, −1.670] (Fig 2E and F), which revealed that high glucose induced the apoptosis of HPCs could be significantly blocked by the overexpression of DJ-1. Caspase 3 is a cysteine protease that plays a central role in apoptosis. Cleaved caspase 3 refers to the activated form of caspase 3, which is generated during apoptosis. Bcl-2 is a crucial protein involved in the regulation of apoptosis, which can primarily protect cells from death by inhibiting apoptosis. We further found that high glucose up-regulated the expression of cleaved caspase 3, and down-regulated the expression of Bcl2. Importantly, overexpression of DJ-1 could block this phenomenon markedly (Fig 2G), suggesting that DJ-1 could attenuate the high glucose-induced apoptosis of HPCs.

### DJ-1 increases the expression of NF-κB and AP-1 by inducing the accumulation of ERK1/2 phosphorylation in HPCs

**Fig 3:**
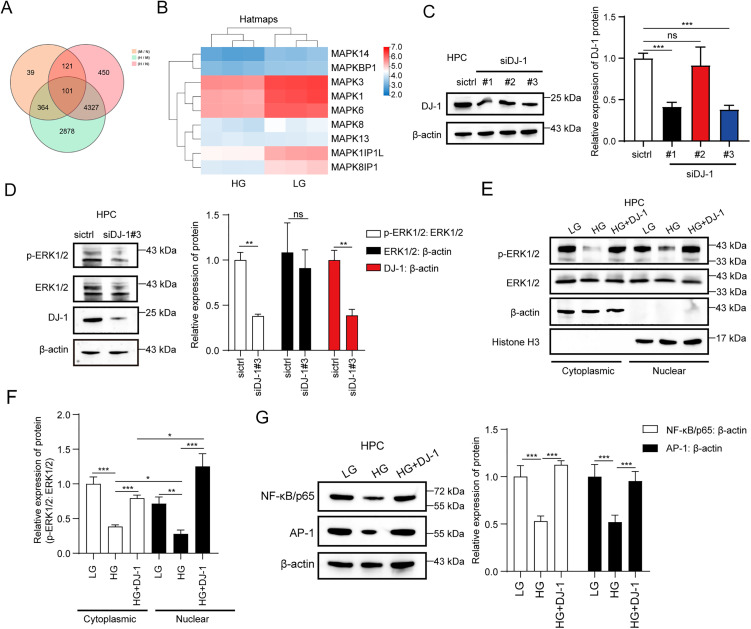
DJ-1 increases the expression of NF-κB and AP-1 by inducing the accumulation of ERK1/2 phosphorylation in HPCs. **(A)** Custom VennMap diagrams were generated by using the RNA-seq data from HPCs treated with LG, MG and HG, respectively. **(B)** Heat map was performed to analyze the MAPK-regulated genes from the 110 changed genes of HPCs with the treatment of LG and HG. **(C)** Representative Western blot images and quantitative statistical bar graphs of protein expression levels of HPCs transfected with siDJ-1#1, siDJ-1#2, siDJ-1#3 were examined by Western blotting analysis and Image J software, respectively. **(D)** Representative Western blot images and quantitative statistical bar graphs of protein expression levels of ERK1/2 and p-ERK1/2 in HPCs transfected with siDJ-1#3 were analyzed by Western blotting analysis and Image J software, respectively. (E) and **(F)** Representative Western blot images and quantitative statistical bar graphs of protein expression levels of ERK1/2 and p-ERK1/2 in HPCs treated with LG, HG and HG + DJ-1 were analyzed by Western blotting analysis and Image J software, respectively. **(G)** Representative Western blot images and quantitative statistical bar graphs of protein expression levels of NF-κB p65 and AP-1 in HPCs treated with LG, HG and HG + DJ-1 were analyzed by Western blotting analysis and Image J software, respectively. MG: mannitol, LG: 5.5mM D-glucose, MG: 15mM D-glucose, HG: 30mM D-glucose, DJ-1: transfected with 1 μg/mL DJ-1-Flag-pcDNA3.1 plasmid. Data are shown as mean ± SD and representative of three independent experiments. *p < 0.05; **p < 0.01; ***p < 0.001.

To clarify the mechanism by which DJ-1 suppresses the high glucose-induced apoptosis of HPCs, we further analyzed the RNA-seq profile. Based on the comparative analysis of the high-glucose (HG) vs. low-glucose (LG) group, medium-glucose (MG) vs. LG group, and HG vs. MG group, we generated a Venn diagram revealing 101 genes with consistent changes across all three groups (Fig 3A), which indicated that these genes exhibit similar expression patterns or changes under different glucose conditions. Notably, among those genes, several MAPK-related genes were changed (Fig 3B). As shown in Fig 3B, the expression of MAPK3 and MAPK1 (also known as ERK1/2) was significantly up-regulated in the lower glucose condition. Thus, we hypothesized that DJ-1 might induce the function of HPCs *via* the ERK1/2 pathway under high glucose conditions. We first synthesized DJ-1-specific siRNAs and verified its knockdown efficiency on DJ-1 expression. The Western blotting analysis showed that siDJ-1#3 revealed the best inhibition efficiency on DJ-1 (Fig 3C). We then used siDJ-1#3 for further analyses. We found that knock-down of DJ-1 by siDJ-1#3 significantly suppressed the phosphorylation level of ERK1/2 (Thr202/Tyr204), and down-regulated the expression of ERK1/2 slightly, in which DJ-1 siRNA reduced the p-ERK1/2: ERK1/2 densitometric ratio by ≈ 62% (0.38 ± 0.02 vs siRNA group: 1.00 ± 0.06, P = 0.0077) (Fig 3D). It has been reported that phosphorylation of ERK1/2 markedly enhances ERK1/2’s activity, enabling it to translocate into the nucleus where it regulates the expression of nuclear target genes. We next explored the function of DJ-1 on p-ERK1/2 nuclear transport [12]. We extracted the nuclear and cytoplasmic proteins of HPCs, respectively. Western blotting analysis showed that high glucose significantly decreased the levels of p-ERK1/2 in both the nucleus and cytoplasm (Fig 3E). Importantly, overexpression of DJ-1 could rescue the levels of p-ERK1/2. Notably, after the overexpression of DJ-1, the levels of p-ERK1/2 were increased relative to low glucose conditions in the nucleus, and this phenomenon was reversed in the cytoplasm (Fig 3E and F), suggesting that DJ-1 could rescue the high glucose-induced inhibition of p-ERK1/2 and induce the transport of p-ERK1/2 from the cytoplasm to the nucleus. It has been reported that p-ERK1/2 can promote the expression of transcription factors NF-κB and AP-1, which in turn inhibit apoptosis [17–19]. Our study found that high glucose inhibits the expression of NF-κB p65 and AP-1. Overexpression of DJ-1 could markedly block this event (Fig 3F). Overall, our study found that DJ-1 increases the expression of NF-κB and AP-1 by inducing the accumulation of ERK1/2 phosphorylation in HPCs.

### Inhibition of ERK1/2 blocks the DJ-1-induced suppression of apoptosis in HPCs

**Fig 4:**
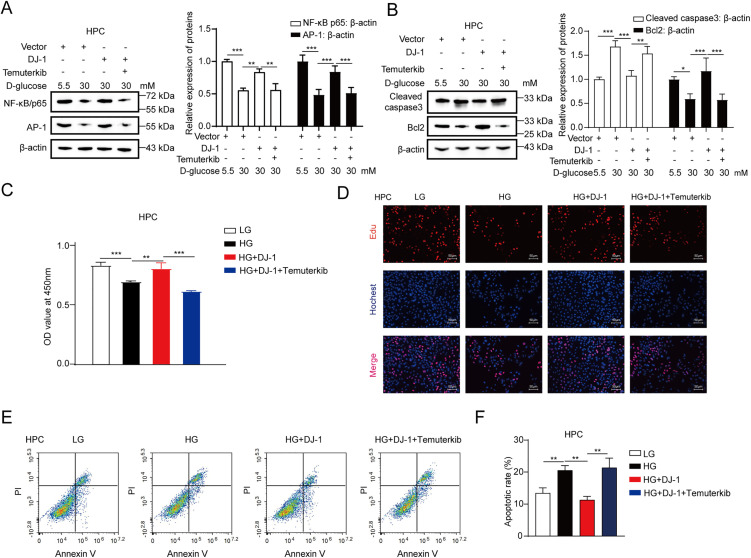
Inhibition of ERK1/2 blocks the DJ-1-induced suppression of apoptosis in HPCs. **(A)** Representative Western blot images and quantitative statistical bar graphs of protein expression levels of NF-κB p65 and AP-1 in HPCs treated with LG, HG, HG + DJ-1 and HG + DJ-1 + temuterkib were analyzed by Western blotting analysis and Image J software, respectively. **(B)** Representative Western blot images and quantitative statistical bar graphs of protein expression levels of cleaved caspase 3 and Bcl2 in HPCs treated with LG, HG, HG + DJ-1 and HG + DJ-1 + temuterkib were analyzed by Western blotting analysis and Image J software, respectively. **(C)** The cell viability was measured by CCK-8 assays in HPCs treated with LG, HG, HG + DJ-1 and HG + DJ-1 + temuterkib, respectively. **(D)** The cell viability was analyzed by EdU assays in HPCs treated with LG, HG, HG + DJ-1 and HG + DJ-1 + temuterkib, respectively. **(E)** The cell apoptosis was analyzed by Annexin V-FITC/PI apoptosis assay in HPCs treated with LG, HG, HG + DJ-1 and HG + DJ-1 + temuterkib, respectively. **(F)** The apoptotic rate of HPCs treated with LG, HG, HG + DJ-1 and HG + DJ-1 + temuterkib were analyzed by GraphPad Prism 8 software. MG: mannitol, LG: 5.5mM D-glucose, MG: 15mM D-glucose, HG: 30mM D-glucose,temuterkib: 1μM, DJ-1: transfected with 1 μg/mL DJ-1-Flag-pcDNA3.1 plasmid. Data are shown as mean ± SD and representative of three independent experiments. *p < 0.05; **p < 0.01; ***p < 0.001.

Next, we explored the function of the DJ-1-ERK1/2 axis on HPC apoptosis. Western blotting analysis showed that temuterkib (LY3214996), an inhibitor of ERK1/2, significantly suppressed the DJ-1-induced up-regulation of NF-κB p65 and AP-1 in the condition of high glucose (Fig 4A). Furthermore, temuterkib could reverse the DJ-1-induced increase in Bcl-2 levels and abolish the DJ-1-induced reduction in cleaved caspase 3 levels (Fig 4B), suggesting that inhibition of ERK1/2 could suppress the DJ-1-induced attenuation of apoptosis-related pathways in HPCs. Next, we performed CCK-8 and EdU assays to study the function of the DJ-1-ERK1/2 axis on podocyte viability. As shown in Fig 4C and D, DJ-1 could enhance the viability of HPCs in the condition of high glucose, and temuterkib could block this phenomenon. In addition, temuterkib could suppress the DJ-1-induced inhibition of podocyte apoptosis under the high glucose conditions (Fig 4E and F), suggesting that inhibition of ERK1/2 blocks the DJ-1-induced suppression of apoptosis in HPCs. Therefore, we conclude that DJ-1 alleviates high glucose-induced podocyte injury *via* activating ERK1/2-NF-κB/AP-1 signaling.

## Discussion

This study reveals that a high-glucose environment downregulates the expression of DJ-1 in HPCs. Overexpression of DJ-1 inhibits podocyte apoptosis by promoting ERK1/2 phosphorylation and its nuclear translocation, thereby activating the transcriptional activity of NF-κB p65 and AP-1. This finding uncovers a novel protective signaling axis (DJ-1/ERK/NF-κB p65/AP-1) mediated by DJ-1 against high-glucose-induced injury.

While previous studies have suggested a protective role for DJ-1 in renal ischemia/reperfusion injury, its specific function and mechanism in diabetic nephropathy, particularly in high glucose-induced podocyte injury, remain unclear [15]. This study is the first to systematically demonstrate the protective role of DJ-1 in a high-glucose-induced podocyte injury model and to elucidate its downstream molecular mechanism in detail. ERK1/2, as key members of the MAPK family, exhibit highly context-dependent functions [13]. In this study, DJ-1-mediated activation of ERK1/2 demonstrated a clear anti-apoptotic effect under acute high-glucose stress. This presents a meaningful contrast to previous findings suggesting that chronic ERK activation may promote fibrosis, indicating that the signaling output of ERK1/2 may vary depending on stimulus intensity, duration, and cell type. The results of this study provide new experimental evidence for understanding the complex roles of ERK1/2 at different stages of diabetic nephropathy and in different cell types.

NF-κB and AP-1 are key transcription factors regulating cell survival and death, and their functions are also highly context-dependent [20,21]. While they often drive apoptosis under chronic inflammation or severe stress, they can promote cell survival under moderate stress or upon activation of protective signals [20,22,23]. Previous studies have reported that RANK-mediated upregulation of NF-κB p65 promotes glomerular oxidative stress and the production of proinflammatory cytokines, thereby exacerbating podocyte injury [24]. Our findings on NF-κB p65 exhibit certain discrepancies with those of this study, which stem fundamentally from the inherent differences in research models, research focuses and upstream regulatory pathways. The aforementioned study used mouse podocytes as the model and focused on RANK-mediated regulation of inflammatory factor expression, in which NF-κB p65 was merely regarded as an intermediate molecule in inflammatory responses without investigating its association with podocyte injury and apoptosis. In contrast, our study adopted terminally differentiated immortalized human podocytes as the research object and centered on exploring the regulatory effect of DJ-1-mediated NF-κB p65 on high glucose-induced podocyte apoptosis, with its direct regulatory role in podocyte apoptosis verified through multi-dimensional experiments. Additionally, in the aforementioned study, NF-κB p65 is activated by RANK-specific downstream signaling, a mode that may exacerbate high glucose-induced podocyte injury by promoting the release of inflammatory cytokines. However, our study confirmed that NF-κB p65 is specifically regulated by the DJ-1/ERK1/2 pathway, and the upregulation of NF-κB p65 mediated by this pathway acts as a protective signal that effectively inhibits podocyte apoptosis. We further evaluated the effects of high glucose on the key factors involved in the non-canonical NF-κB signaling pathway in HPCs by using RNA-seq profiling. We found that high glucose had no significant effect on most of the non-canonical NF-κB signaling-related factors, with the exception of CHUK (IKKα) (data not shown). These results indicate that high glucose treatment can upregulate the expression of IKKα, a core component of the non-canonical NF-κB signaling pathway, which may also contribute to high glucose-mediated HPC injury. Whether DJ-1 can regulate proinflammatory cytokine secretion and podocyte injury by targeting IKKα remains to be further investigated. Our finding extends the function of DJ-1 from a known sensor of oxidative stress to a coordinator of transcriptional regulatory networks, deepening the understanding of DJ-1-mediated cytoprotective mechanisms.

The DJ-1/ERK axis identified in this study provides a novel mechanistic addition to podocyte protection. Although prior research has hinted at DJ-1 influencing podocytes via the PTEN pathway [15], this study clearly reveals the direct pathway through which DJ-1 regulates NF-κB/AP-1 via ERK1/2. This positioning places DJ-1 at a critical node within the podocyte injury response network, where its loss of function may lead to failure in protective signal transduction, thereby exacerbating apoptosis. From a translational perspective, maintaining or restoring DJ-1 function in podocytes, particularly in the early stages of diabetic nephropathy, could represent a potential strategy to enhance podocyte resilience. Targeting the DJ-1/ERK axis may offer a novel synergistic approach complementary to existing therapies (e.g., SGLT2 inhibitors), providing new avenues for delaying the progression of diabetic nephropathy. It is well established that high glucose-induced inflammatory activation plays a critical role in podocyte injury during diabetic kidney disease. In line with this, our RNA-seq analysis revealed that high glucose treatment significantly upregulated the expression of multiple proinflammatory cytokines and chemokines in HPCs, including CXCL8, IL-1α, IL-1β and IL-6 (data not shown). These results further validate that high glucose can effectively trigger an inflammatory response in HPCs and contribute to podocyte dysfunction and damage. Nevertheless, whether DJ-1 is implicated in this process by modulating the expression of the above inflammatory mediators remains to be further elucidated in subsequent studies.

Certainly, this study has limitations. The conclusions are primarily based on in vitro experiments using a single immortalized human podocyte line. Future validation using multiple primary podocyte cell lines, DJ-1 knockout animal models, or spontaneous diabetic nephropathy models (e.g., db/db mice) is needed to strengthen the generalizability of these findings. Furthermore, questions such as whether DJ-1 expression levels could serve as a biomarker for risk stratification in diabetic nephropathy and the specific spectrum of downstream target genes it regulates warrant further exploration.

In summary, this study not only confirms the phenomenon of high glucose downregulating DJ-1 expression in HPCs, but more importantly, elucidates a molecular pathway driven by DJ-1 that exerts anti-apoptotic effects by activating ERK1/2 and directing NF-κB/AP-1 activity. This work moves beyond a simple description of DJ-1 expression changes, providing a novel and specific mechanistic explanation for its protective function in podocyte injury during diabetic nephropathy. It offers important theoretical foundations for understanding the pathophysiology of this disease and exploring new intervention strategies.

## Supporting information

S1 File Raw images. (PDF)

S2 File Supplemental File. (XLS)
