# Supplementary figures and images for: DJ-1 alleviates high glucose-induced podocyte injury via activating ERK1/2 signaling
Source: PLoS One. 2026 Apr 17;21(4):e0346714. doi: 10.1371/journal.pone.0346714 (PMC13089688; doi:10.1371/journal.pone.0346714)

Raw data of figure 2

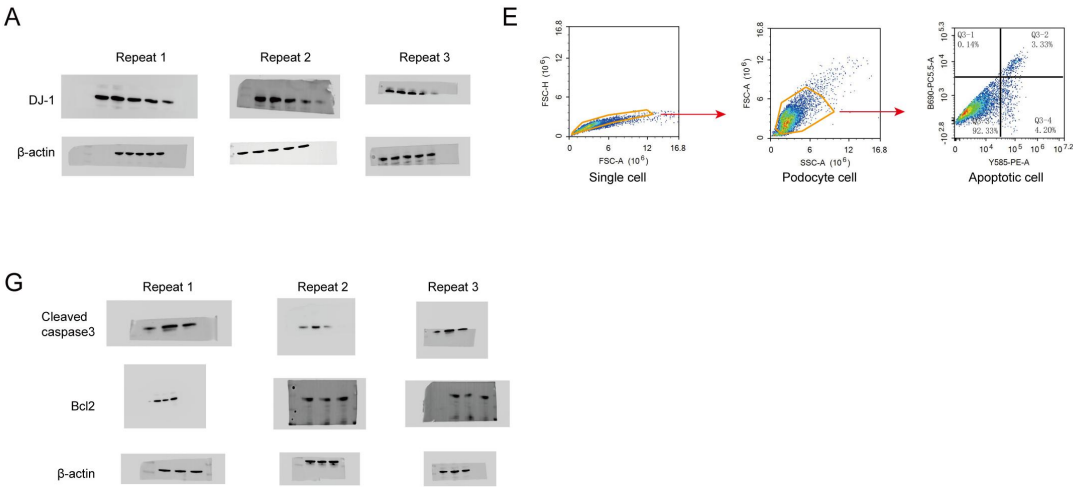

Raw data of figure 3

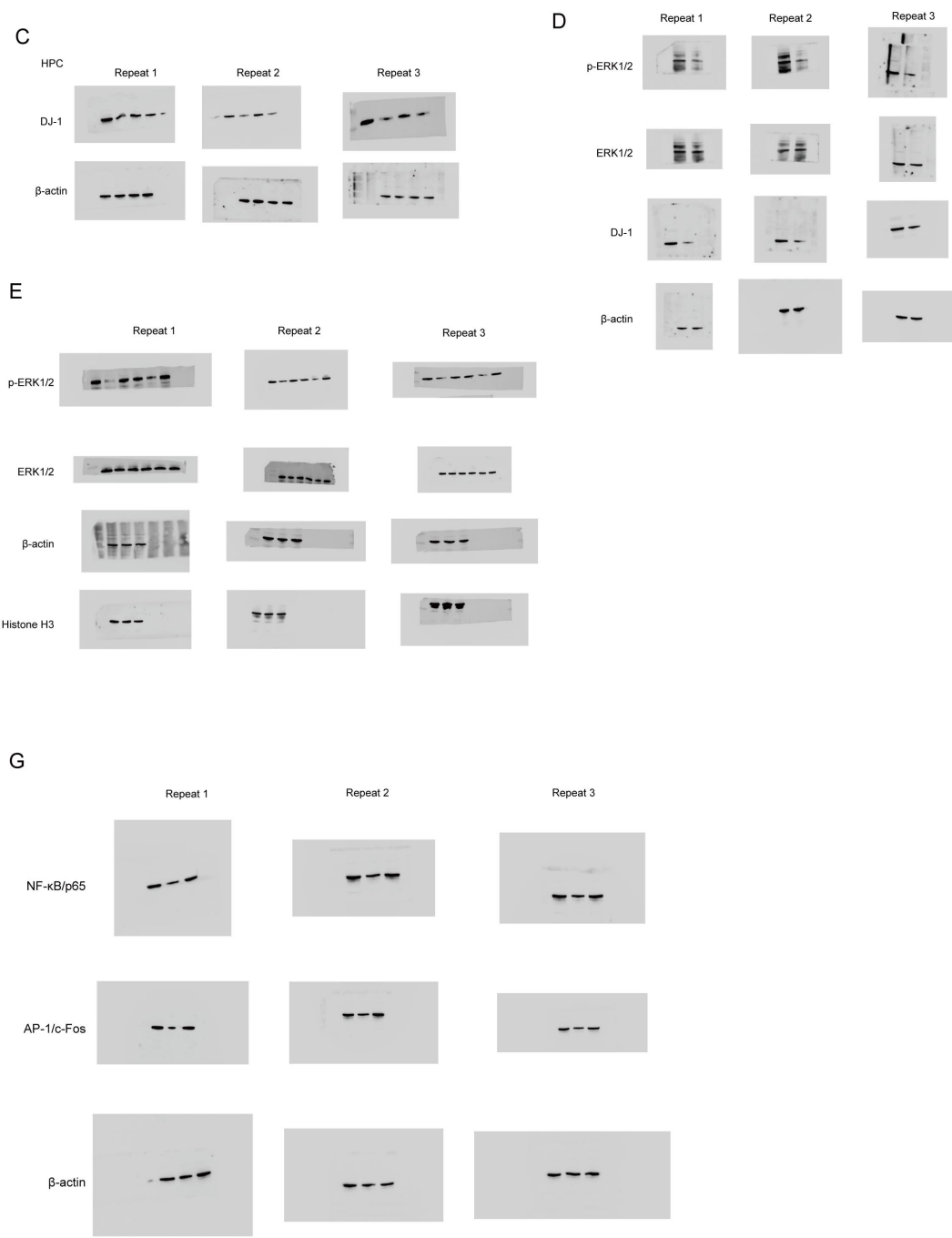

Raw data of figure 4

A

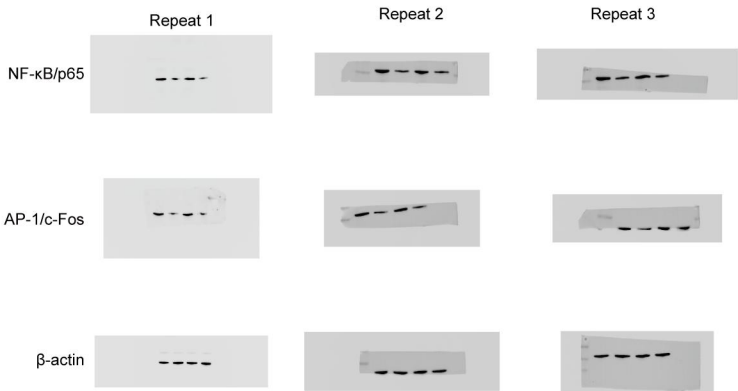

B

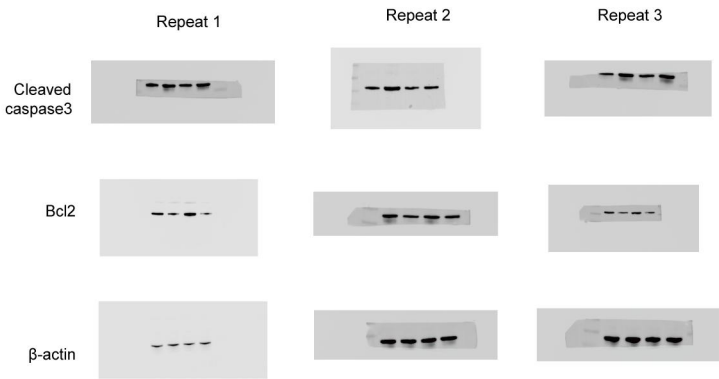

Supplement: S1 File — (PDF) [file pone.0346714.s001.pdf]
